# Beyond the Impact Factor – What do alternative metrics have to offer?

**DOI:** 10.3205/zma001104

**Published:** 2017-05-15

**Authors:** Götz Fabry, Martin R. Fischer

**Affiliations:** 1 Albert-Ludwig-Universität Freiburg, Abt. für Med. Psychologie, Freiburg/Brg, Germany; 2 GMS Journal for Medical Education, Assistant Chief Editor, Erlangen, Germany; 3 Klinikum der Universität München, Institut für Didaktik und Ausbildungsforschung in der Medizin, München, Germany; 4 GMS Journal for Medical Education, Chief Editor, Erlangen, Germany

## Editorial

Some of our readers might have already noticed that lately some of the articles in the JME are marked with a “donut,” a ring composed of colored, intertwined rings around a number at its center. It is the emblem of Altmetric.com [http://www.altmetric.com] a company named after the general term for indicators measuring the dissemination of scientific literature beyond the Impact Factor (“alternative metrics” or “altmetrics”) [1], [2]. The donut indicates which online media refer to the respective article. The greater the number of colors in the ring, the greater the number of different media linked to the article. General news sites and newspapers, the scientific bibliographic platform Mendeley, sites for post-publication peer review (e.g. Publons, see below), references in Scopus (a bibliographic database run by Elsevier), Wikipedia, blogs, social media like Facebook and Twitter, YouTube, and a multitude of other resources are analyzed. The number in the center of the ring is the “attention score,” a weighted measure to represent the coverage of the respective article in the media analyzed (for more details on how the score is calculated, see https://goo.gl/jSLn1Y and [3]). A click on the emblem leads to a website that reports the exact details and the geographical distribution of the media-related activities.

What is the relevance of this kind of analysis and of the alternative score? First of all, it expresses a change in scientific communication, albeit a very slow and time-delayed one [4]. Currently, the productivity of scientists and the quality of their work are mainly measured by their publications. Despite the fact that the digital revolution permeates all aspects of our daily lives, the form of these publications has remained remarkably constant. Scientific evidence is still published in journal articles that are, in many cases, still organized in volumes and issues with page numbers even though they are only rarely printed on paper, at least in medicine and the natural sciences. Furthermore, the system of gratification for scientific achievement relies on these structures, too. The number of articles with very high impact remains of crucial importance for a career in science. To date, this impact is measured almost exclusively by the Impact Factor, a measure that specifies how often articles in a given journal are referenced by other scientific journals within the previous two years [5]. The use of the Impact Factor as a means to evaluate research quality especially with regard to individuals has been increasingly criticized. The San Francisco Declaration on Research Assessment (DORA), launched in 2012 and so far signed by more than 500 organizations and 12,000 scientists, states that the Impact Factor and similar journal metrics should not be used to assess the quality of individual articles or their authors, nor for decisions on hiring, promotion and tenure [http://www.ascb.org/dora/], [6]. However, there is no sign that a fundamental change will take place in the foreseeable future, despite the fact that a number of alternative strategies exist regarding the different aspects of traditional publishing conventions.

In recent years, for instance, “post-publication peer review” (PPPR) has taken root as an alternative to the usual “pre-publication peer review” [7]. The reasons are multifaceted. First of all, there is almost no evidence that traditional peer review actually increases the quality of manuscripts, in part due to the methodological challenges that can handicap research [8]. A recently published study examined this issue by looking at journals of the BMC platform since these journals publish an article’s “pre-publication history,” meaning all reviews and author responses [9]. The results show that the reviewers made few suggestions for changes overall. While the majority of these suggestions improved the quality of the manuscripts, there were also some suggestions that decreased the quality. Moreover, the reviewers missed many flaws and errors in the manuscripts that would have been relatively easy to detect by using simple tools such as a checklist based on the CONSORT statement [10]. Further criticism refers to delayed publication caused by the pre-publication peer review and to the fact that the review process usually involves only two or three reviewers whose expertise is not always known or transparent [7].

PPPR is intended to overcome these weaknesses. An example from the field of medical education is “AMEE MedEdPublish” [www.mededpublish.org] which went online very recently. Manuscripts are published immediately without prior review or, as is the case with MedEdPublish, after a more formal check by the editorial office. Readers review the manuscripts after publication. While this procedure generally can take many forms, MedEdPublish allows anyone who is registered on the platform to review and rate the article. The hope behind this rationale is that by involving a potentially unlimited number of reviewers this will increase the reliability and validity of the review. However, there is also a panel of official reviewers whose judgment is essential on whether or not an article receives a recommendation. It is the intention of the initiators that this recommendation will then lead to referencing the respective article in PubMedCentral which, in turn, would put it on par with publications from PubMed-indexed journals with traditional peer review. In some places, at least, this would also make it count toward career advancement.

While this kind of PPPR is intended to replace the traditional peer review, the term also encompasses all kind of criticism and comments relating to an article after publication, even when the article has undergone the usual peer review. Publication scandals that occur time and again make it quite clear that this is indeed necessary. Flaws in publications on stem cells in highly ranked journals, for instance, were discovered by well-established bloggers and resulted in a retraction of the affected papers [11]. Letters to the editor are in fact a long-standing possibility to criticize or comment on an article but compared to the potential of the internet, this type of scientific communication seems rather old-fashioned. If letters are published, they are often released with considerable delay and they do not always lead to a reply by the authors, let alone a reaction by the journals who – on top of it all – have a conflict of interest when it comes to publishing critical letters [12].

In light of this, it seems obvious that the internet be used to critique and discuss scientific publications. However, what appears to be self-evident is not as trivial as it may seem: where and how, exactly, should these discussions and commentaries take place? How, for instance, to avoid the well-known difficulties of communication within social networks, e.g. polemics or hyper-criticism under the cover of anonymity or fake profiles? Who is going to participate in this discussion? What kind of motives will prompt the participants?

With regard to the site of the discussion, solutions are already apparent in the form of social networks for scientists, such as ResearchGate [www.researchgate.net] and Academia [www.academia.edu]. On these platforms scientists can share their publications, read and comment on the publications of others, and engage in discussions on different topics, as in other internet forums. On ResearchGate these activities, along with the responses they trigger, are registered by calculating a specific measure (“RG score”) that – at least in the company’s view – mirrors the reputation of the individual scientist within the platform and, perhaps, even beyond. Studies examining ResearchGate revealed that, for now, the social and network-related functions are rarely used except to share scientific articles (although these have to be uploaded first which is not without problems in terms of copyright) [13]. An interesting question in this regard is whether the number of “reads” on ResearchGate (which is also one of the components of the RG score) correlates with other measures, for instance, the number of citations. A recent study showed that “younger” articles outnumber “older” articles on the platform, and that younger articles tend to be read more often. A comparison of reads on ResearchGate with the number of citations in the database Scopus and the number of readers on the bibliographic platform Mendeley resulted in rather low correlations which, according to the authors of the study, might indeed indicate different target audiences [14]. This could mean that the different measures of the different media and platforms do indeed measure different aspects regarding the dissemination of and the response to science which would justify their respective uses [15].

However popular ResearchGate, Academia and Mendeley might be by now, it remains unsatisfactory from a scientific perspective that all these platforms are run by privately owned companies pursuing their own opaque, commercial agendas. In this context another initiative appears to be much more promising: in 2013 PubMed launched “PubMed Commons” a project that is open to everyone who has authored at least one publication listed in PubMed. The service can be used via the free individual NCBI account. This opens up the possibility to comment on each article indexed in PubMed. The box for the comments is placed right under the article abstract in PubMed, and the comments are visible to everyone who retrieves the article in PubMed. Thus, all articles of the JME (and the ZMA) published since 2005 are also open for comments and discussion in this manner. Evidently, this option has not yet been used, and generally the intensity of the exchange on PubMed Commons is rather modest. The reasons for this are obvious: due to a severe lack of time, only very few scientists will feel the necessity to become active, especially since there is no reward terms of career advancement aside from the fulfillment of idea-driven motives and scientific interest that might pay off it. Additional reasons that are generally known from social networks might also be relevant. Particularly scientists at the beginning of their career might question whether publicly criticizing a paper written by an established colleague might impair future career opportunities. In addition, social dynamics might have a negative impact in terms of stereotyping or gender bias [16]. These considerations illustrate that PPPR, too, might have drawbacks which is one reason why the JME currently retains the blinded pre-publication peer review.

At least with regard to the reward for preparing a scholarly peer review (pre- and post-publication), solutions are also being developed. Reviewers can create an account on the “Publons” portal [publons.com] to publicly document their activity as reviewers. Since a number of big publishers (Springer, Thieme, BMJ, etc.) support the portal, reviewers are asked to document their reviews on Publons as part of the routine reviewing process. Depending on the type of contract with the journals, different modes of documentation exist ranging from simple and anonymous documentation of the number of completed reviews to the full-text documentation of the review itself. Even if a publisher or journal has no contract with Publons yet, as is currently the case for JME, reviews can still be documented quantitatively. Publons has also installed an index measuring the quantity and quality of the reviews. It is the idea of the Publon founders that this measure might pay off in terms of career advancement or at least academic recognition [17].

This cursory overview on some of the online activities that contribute to the Altmetric donut and score makes it clear that a multitude of opportunities for public scientific communication exist beyond journal articles and the heavily criticized Impact Factor. Currently, their potential has not yet been fully explored, and their significance cannot be fully appraised [18]. However, it seems necessary and reasonable to track these activities and to actively contribute to them as far as individual prospects and resources allow. In this light, we invite all of our authors, reviewers and readers to refer to their work in alternative media and networks to intensify our professional discourse and, not least, to increase the attention given to the JME [19].

## Competing interests

The authors declare that they have no competing interests.
